# Commentary: SEMA3B is associated with disease activity and infliximab response in IBD patients but does not contribute to the development of intestinal inflammation *in vivo*

**DOI:** 10.3389/fimmu.2026.1836065

**Published:** 2026-06-05

**Authors:** Yang Yu, Liang Zhan, Zhenzhen Tang

**Affiliations:** 1 Department of Gastroenterology, Suining Central Hospital, Suining, China; 2 Department of Neuroelectrophysiology, Suining Central Hospital, Suining, China

**Keywords:** biomarker, disease activity, inflammatory bowel disease, infliximab, SEMA3B

## 1 Introduction

The marked heterogeneity of inflammatory bowel disease (IBD) makes it unlikely that any single molecule can serve simultaneously as a robust indicator of disease activity, a reliable predictor of therapeutic response, and a direct mediator of intestinal inflammation (1). In this context, the recent study by Arosa et al. (2) is of considerable interest. By combining transcriptomic analyses of multiple patient cohorts with *in vivo* experiments in a dextran sulfate sodium (DSS)-induced colitis model, the authors reported that SEMA3B is associated with disease activity and infliximab response in IBD, but does not appear to directly contribute to the development of intestinal inflammation *in vivo*.

A notable strength of this work lies in its cautious interpretation. Rather than extending clinical associations directly into therapeutic relevance, the authors examined the functional role of SEMA3B *in vivo* and arrived at a restrained conclusion. This strengthens the overall credibility of the study. At the same time, several aspects of the findings deserve further discussion. In particular, it remains unclear whether SEMA3B should be viewed primarily as a surrogate marker of mucosal inflammatory status, a stratification signal for treatment response, or a functional molecule whose role was not fully captured by the experimental setting. This commentary is based on the published findings of Arosa et al. and does not include an independent re-analysis of the original transcriptomic datasets or primary experimental data. Therefore, our interpretation should be viewed as a conceptual and translational discussion rather than a validation study.

SEMA3B belongs to the class 3 semaphorin family, members of which have been implicated in immune regulation, cell migration, epithelial–stromal communication, angiogenesis, and tissue remodeling (3–5). Previous studies have also suggested that SEMA3B may exert anti-inflammatory and regulatory effects in immune-mediated diseases, including rheumatoid arthritis (6). In the intestinal context, these biological processes are closely related not only to inflammatory activation but also to mucosal repair and barrier restoration. Therefore, SEMA3B may be better interpreted within a dynamic mucosal microenvironment rather than as a single pro- or anti-inflammatory molecule.

## 2 SEMA3B as a stratification marker

The main value of this study may not lie in determining whether SEMA3B is pathogenic *per se*, but in underscoring a broader point in IBD research: molecules associated with disease activity or treatment response are not necessarily direct drivers of disease progression. Arosa et al. showed that SEMA3B expression was reduced in intestinal tissue from patients with IBD and correlated with disease activity. They also found baseline differences in SEMA3B and related receptor molecules between infliximab responders and non-responders. However, the predictive performance was modest, suggesting that SEMA3B may reflect one component of the inflammatory microenvironment rather than serve as a robust standalone biomarker.

This distinction is relevant to precision medicine in IBD. Biomarkers with clinical value are increasingly viewed not as isolated molecules, but as components of broader molecular networks. In this context, the significance of SEMA3B may lie less in independent prediction than in its potential contribution to composite models that identify inflammatory phenotypes more likely to benefit from anti-TNF therapy.

Another point that should be considered is whether SEMA3B can be evaluated as a non-invasive biomarker. The original study primarily relied on tissue transcriptomic data, and circulating or fecal SEMA3B levels remain insufficiently characterized. Future studies assessing serum or fecal SEMA3B may help determine whether this molecule has practical value for clinical monitoring.

## 3 Interpreting the association with infliximab response

The authors (2) suggest that SEMA3B may be more closely linked to infliximab response than to clinical remission overall. This is an interesting observation, but it warrants cautious interpretation. The biologic-treated cohorts differed in patient characteristics, sampling sites, transcriptomic platforms, outcome definitions, and the timing of response assessment, all of which may influence the association between baseline gene expression and clinical outcome. Thus, the current evidence may support an association between SEMA3B and response in certain anti-TNF treatment settings, rather than a strictly drug-specific mechanistic role.

Moreover, response to anti-TNF therapy likely reflects a broader inflammatory context rather than the action of a single agent alone. SEMA3B expression may be shaped by TNF signaling, mucosal injury, and local cell–cell communication. Therefore, it may reflect a mucosal state that is more permissive to anti-TNF benefit, rather than a mechanism unique to infliximab. This distinction is important for future translational studies.

In addition, disease location, such as colonic versus ileal Crohn’s disease, concomitant medications, and baseline inflammatory burden, may confound the observed association between SEMA3B expression and infliximab response. Sex-related differences should also be considered because semaphorin signaling may have sex-dimorphic effects in inflammatory settings. These factors should be incorporated into future prospective studies before a drug-specific interpretation can be established.

The findings related to golimumab and vedolizumab should also be interpreted in this context. If SEMA3B shows a similar association with golimumab, another anti-TNF agent, this may support the idea that SEMA3B reflects an anti-TNF-responsive mucosal inflammatory state rather than an infliximab-specific mechanism. By contrast, a weaker or inconsistent association with vedolizumab may suggest that SEMA3B is less related to lymphocyte trafficking blockade and more closely linked to TNF-associated mucosal inflammation. However, because these cohorts differed in design, treatment background, sampling strategy, and outcome definition, direct comparison across biologics should remain cautious.

## 4 Interpreting the negative *in vivo* findings

In the DSS-induced acute colitis model, recombinant SEMA3B did not clearly improve disease severity, leading the authors to conclude that SEMA3B does not contribute to the development of intestinal inflammation *in vivo*. While this interpretation is reasonable, the negative findings may warrant a more cautious reading. The DSS model primarily reflects acute epithelial injury and innate immune-driven inflammation, making it more suitable for assessing molecules involved in disease initiation than those acting during resolution, tissue repair, or microenvironmental remodeling. It therefore remains possible that SEMA3B influences mucosal recovery rather than inflammatory onset. As the authors acknowledge, the study was also limited by the use of a single dose, the absence of circulating SEMA3B measurements, and a focus on the early phase of disease. Together, these considerations suggest that further evaluation in more specific chronic or repair-phase models will be necessary to clarify whether SEMA3B exerts context-dependent effects not captured in acute DSS colitis. Potential models may include repeated-cycle DSS colitis to mimic relapsing chronic inflammation, DSS withdrawal/recovery models to assess mucosal healing after epithelial injury, TNBS-induced chronic colitis to evaluate persistent immune-mediated inflammation, and IL-10 knockout spontaneous colitis to study SEMA3B under conditions of chronic immune dysregulation. In addition, intestinal organoid-based wound-healing models may help determine whether SEMA3B directly affects epithelial repair, barrier restoration, or epithelial–stromal communication.

It should also be acknowledged that negative *in vivo* findings may be underrepresented in the literature because of publication bias. In this regard, the study by Arosa et al. is valuable because it reports a functionally negative result rather than forcing a mechanistic conclusion from clinical associations.

## 5 Moving from a single-molecule model to a network view

Another notable observation is that recombinant SEMA3B administration was accompanied by coordinated changes in the expression of several semaphorin family members and related receptors. This finding suggests that SEMA3B acts within a dynamic regulatory network rather than along a simple linear pathway. In the adaptive mucosal immune environment of IBD, modulation of a single ligand may trigger broader reorganization of ligand–receptor interactions, potentially offsetting or reshaping the expected biological effect.

For this reason, a strictly linear framework based on single-gene differential expression, single-protein intervention, and single-endpoint assessment may be insufficient to define the role of SEMA3B. A network-based interpretation centered on semaphorin reprogramming, cellular communication, and microenvironmental adaptation may better explain why SEMA3B shows clinical associations while lacking an obvious therapeutic effect in an acute *in vivo* model.

## 6 Discussion

Overall, the study by Arosa et al. provides the IBD field with an informative negative result. SEMA3B appears to be associated with disease activity and infliximab response, yet it does not show a clear disease-modifying effect in acute DSS colitis. The importance of this finding lies in its ability to distinguish among three different levels of interpretation: an associated molecule, a mechanistic mediator, and a druggable target.

At present, SEMA3B may be more appropriately regarded as a candidate marker of mucosal inflammatory status and treatment-response stratification than as an established pathogenic factor or therapeutic target. However, this commentary has several limitations. It is based on the published report and does not include independent re-analysis of public datasets or primary data. In addition, the potential influence of disease location, concomitant therapies, sex differences, and non-invasive SEMA3B measurements remains insufficiently addressed. Future studies integrating prospective clinical cohorts, single-cell and spatial omics, and well-defined chronic inflammation, recovery-phase, and epithelial repair models will be important for defining its cellular origin, temporal dynamics, and regulatory context. Rather than asking only whether SEMA3B suppresses acute inflammation, future work should also examine whether it participates in inflammation resolution, epithelial regeneration, and mucosal microenvironment remodeling. Its eventual value may lie not in isolation, but as part of a broader molecular framework for patient stratification in IBD.
